# Bis(2-amino-3-methyl­pyridine-κ*N* ^1^)dichloridomercury(II)

**DOI:** 10.1107/S1600536812032126

**Published:** 2012-07-21

**Authors:** Azadeh Tadjarodi, Keyvan Bijanzad, Behrouz Notash

**Affiliations:** a Department of Chemistry, Iran University of Science and Technology, Tehran 16846-13114, Iran; b Department of Chemistry, Shahid Beheshti University, G. C., Evin, Tehran 1983963113, Iran

## Abstract

In the title compound, [HgCl_2_(C_6_H_8_N_2_)_2_], the two independent Hg^II^ cations are each located on a twofold rotation axis and coordinated by two pyridine N atoms from two 2-amino-3-methyl­pyridine ligands and two Cl^−^ anions in a distorted tetra­hedral geometry. An intra­molecular N—H⋯Cl hydrogen bond occurs in each independent complex mol­ecule. Inter­molecular N—H⋯Cl hydrogen bonds occur in the crystal structure.

## Related literature

For coordination modes of 2-amino-3-methyl­pyridine (ampy), see: Arab Ahmadi *et al.* (2011 ▶); Tadjarodi *et al.* (2010 ▶); Amani Komaei *et al.* (1999 ▶); Ziegler *et al.* (2000 ▶); Castillo *et al.* (2001 ▶); Chen *et al.* (2005 ▶). For proton-transfer compounds incorporating ampy, see: Carnevale *et al.* (2010 ▶).
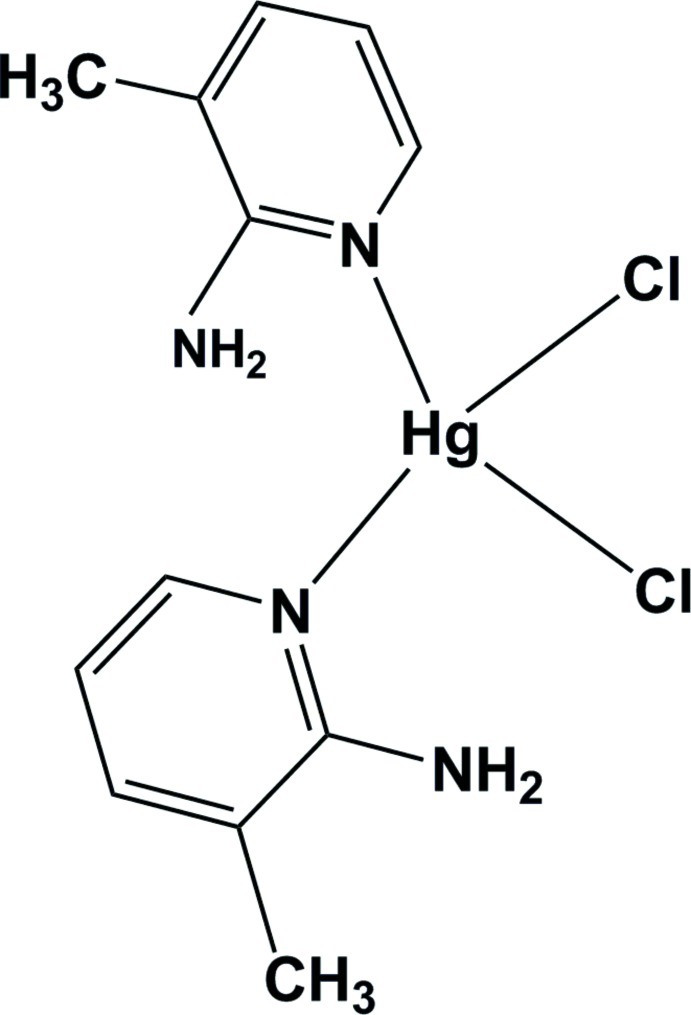


## Experimental

### 

#### Crystal data

[HgCl_2_(C_6_H_8_N_2_)_2_]
*M* *_r_* = 487.78
Monoclinic, 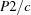

*a* = 16.495 (3) Å
*b* = 6.6320 (13) Å
*c* = 16.273 (3) Å
β = 119.56 (3)°
*V* = 1548.5 (7) Å^3^
*Z* = 4
Mo *K*α radiation
μ = 10.28 mm^−1^
*T* = 298 K
0.30 × 0.30 × 0.27 mm

#### Data collection

Stoe IPDS 2T diffractometer
Absorption correction: numerical [shape of crystal determined optically (*X-SHAPE* and *X-RED32*; Stoe & Cie, 2005 ▶)] *T*_min_ = 0.149, *T*_max_ = 0.168
5454 measured reflections
2912 independent reflections
2493 reflections with *I* > 2σ(*I*)
*R*_int_ = 0.052

#### Refinement

*R*[*F* ^2^ > 2σ(*F* ^2^)] = 0.047
*wR*(*F* ^2^) = 0.116
*S* = 1.03
2912 reflections
188 parameters
4 restraints
H atoms treated by a mixture of independent and constrained refinement
Δρ_max_ = 2.40 e Å^−3^
Δρ_min_ = −2.67 e Å^−3^

### 

Data collection: *X-AREA* (Stoe & Cie, 2005 ▶); cell refinement: *X-AREA*; data reduction: *X-RED32* (Stoe & Cie, 2005 ▶); program(s) used to solve structure: *SHELXS97* (Sheldrick, 2008 ▶); program(s) used to refine structure: *SHELXL97* (Sheldrick, 2008 ▶); molecular graphics: *ORTEP-3 for Windows* (Farrugia, 1997 ▶); software used to prepare material for publication: *WinGX* (Farrugia, 1999 ▶).

## Supplementary Material

Crystal structure: contains datablock(s) I, global. DOI: 10.1107/S1600536812032126/xu5594sup1.cif

Structure factors: contains datablock(s) I. DOI: 10.1107/S1600536812032126/xu5594Isup2.hkl

Additional supplementary materials:  crystallographic information; 3D view; checkCIF report

## Figures and Tables

**Table 1:** Hydrogen-bond geometry (Å, °)

*D*—H⋯*A*	*D*—H	H⋯*A*	*D*⋯*A*	*D*—H⋯*A*
N2—H2*A*⋯Cl1	0.86 (2)	2.58 (4)	3.420 (9)	164 (10)
N2—H2*B*⋯Cl2^i^	0.87 (2)	2.72 (7)	3.420 (8)	139 (8)
N4—H4*A*⋯Cl1^ii^	0.86 (2)	2.70 (7)	3.409 (8)	140 (9)
N4—H4*B*⋯Cl2	0.87 (2)	2.59 (4)	3.424 (9)	162 (9)

Symmetry codes: (i) 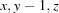; (ii) 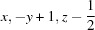.

**Fig. 1.**
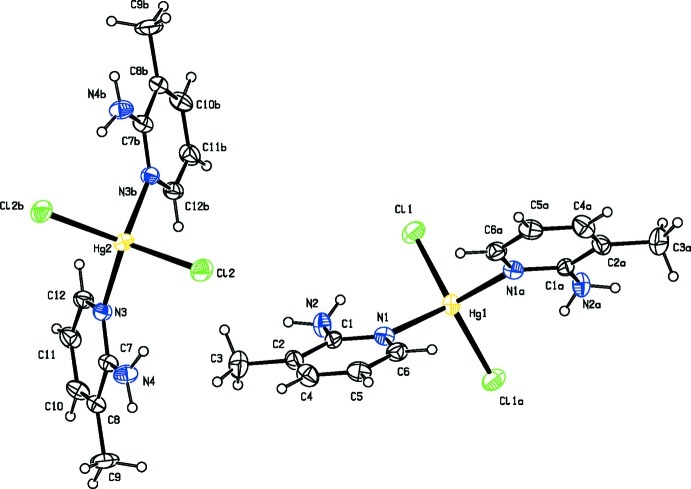
The molecular structure of [Hg(ampy)2Cl2] with displacement ellipsoids drawn at 30% probability level.

**Fig. 2.**
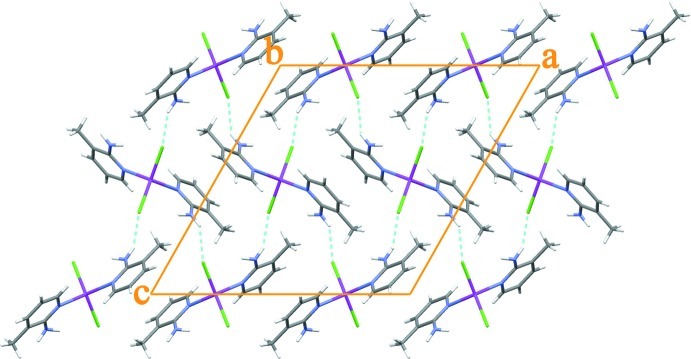
The packing diagram of the title compound showing hydrogen bonding as blue dashed lines.

## supplementary crystallographic information

### Comment

2-Amino-3-methylpyridine (ampy) is capable of coordinating to metals not only through the nitrogen atom of the pyridyl group (Arab Ahmadi *et al.*, 2011; Tadjarodi *et al.*, 2010; Amani Komaei *et al.*, 1999; Ziegler *et al.*, 2000; Castillo *et al.*, 2001) but also *via* the nitrogen atom of the amino group (Chen *et al.*, 2005). So far, different structures of proton-transfer compounds, [(ampyH)_2_Co*X*_4_] (*X* = Cl, Br) have been reported (Carnevale *et al.* (2010).

We report herein the synthesis and molecular structure of the title compound, [Hg(ampy)_2_Cl_2_]. The asymmetric unit of the title compound consists of two half of one mercury, one ampy and one chloride atom. The coordination sphere of the mononuclear complex consists of two chloride ions and two pyridyl nitrogen atoms from two ampy ligands in a distorted tetrahedral geometry (Fig. 1). In the crystal structure of [Hg(ampy)_2_Cl_2_], there are several intermolecular N–H···Cl hydrogen bond interactions which stabilized crystal structure (Fig. 2 & Table 1).

### Experimental

An ethanolic solution of 2-amino-3-methylpyridine (10 mmol) was added to a solution of HgCl_2_ (5 mmol) in ethanol (10 ml) and stirred for 10 min at 50°C. Slow evaporation of the resulting filtrate gave the colorless crystals suitable for X-ray analysis (decomposition > 240 °C).

### Refinement

Hydrogen atoms attached to nitrogen atoms were found in difference Fourier map. H2A, H2B, H4A and H4B were refined with *U*iso(H) = 1.5 *U*eq(N). H2A, H2B, H4A and H4B were refined with distance restraints of N—H 0.86 (2), 0.87 (2), 0.86 (2) and 0.87 (2), respectively. H atoms attached to C atoms were positioned geometrically and refined as riding atoms, with C—H = 0.93 Å (CH), with C—H = 0.96 Å (CH_3_), and *U*_iso_(H) = 1.2,1.5*U*_eq_(C).

### Crystal data

**Table:**

[HgCl_2_(C_6_H_8_N_2_)_2_]	*F*(000) = 920
*M**_r_* = 487.78	*D*_x_ = 2.092 Mg m^−^^3^
Monoclinic, *P*2/*c*	Mo *K*α radiation, λ = 0.71073 Å
Hall symbol: -P 2yc	Cell parameters from 2912 reflections
*a* = 16.495 (3) Å	θ = 2.5–26.0°
*b* = 6.6320 (13) Å	µ = 10.28 mm^−^^1^
*c* = 16.273 (3) Å	*T* = 298 K
β = 119.56 (3)°	Block, colorless
*V* = 1548.5 (7) Å^3^	0.30 × 0.30 × 0.27 mm
*Z* = 4	

### Data collection

**Table:**

Stoe IPDS 2T diffractometer	2912 independent reflections
Radiation source: fine-focus sealed tube	2493 reflections with *I* > 2σ(*I*)
Graphite monochromator	*R*_int_ = 0.052
rotation method scans	θ_max_ = 26.0°, θ_min_ = 2.5°
Absorption correction: numerical [shape of crystal determined optically (*X-SHAPE* and *X-RED32*; Stoe & Cie, 2005)]	*h* = −16→20
*T*_min_ = 0.149, *T*_max_ = 0.168	*k* = −7→8
5454 measured reflections	*l* = −19→20

### Refinement

**Table:**

Refinement on *F*^2^	Secondary atom site location: difference Fourier map
Least-squares matrix: full	Hydrogen site location: inferred from neighbouring sites
*R*[*F*^2^ > 2σ(*F*^2^)] = 0.047	H atoms treated by a mixture of independent and constrained refinement
*wR*(*F*^2^) = 0.116	*w* = 1/[σ^2^(*F*_o_^2^) + (0.0773*P*)^2^] where *P* = (*F*_o_^2^ + 2*F*_c_^2^)/3
*S* = 1.03	(Δ/σ)_max_ < 0.001
2912 reflections	Δρ_max_ = 2.40 e Å^−^^3^
188 parameters	Δρ_min_ = −2.67 e Å^−^^3^
4 restraints	Extinction correction: *SHELXL97* (Sheldrick, 2008), Fc^*^=kFc[1+0.001xFc^2^λ^3^/sin(2θ)]^-1/4^
Primary atom site location: structure-invariant direct methods	Extinction coefficient: 0.0064 (5)

### Special details

**Table:**

Geometry. All e.s.d.'s (except the e.s.d. in the dihedral angle between two l.s. planes) are estimated using the full covariance matrix. The cell e.s.d.'s are taken into account individually in the estimation of e.s.d.'s in distances, angles and torsion angles; correlations between e.s.d.'s in cell parameters are only used when they are defined by crystal symmetry. An approximate (isotropic) treatment of cell e.s.d.'s is used for estimating e.s.d.'s involving l.s. planes.
Refinement. Refinement of *F*^2^ against ALL reflections. The weighted *R*-factor *wR* and goodness of fit *S* are based on *F*^2^, conventional *R*-factors *R* are based on *F*, with *F* set to zero for negative *F*^2^. The threshold expression of *F*^2^ > σ(*F*^2^) is used only for calculating *R*-factors(gt) *etc*. and is not relevant to the choice of reflections for refinement. *R*-factors based on *F*^2^ are statistically about twice as large as those based on *F*, and *R*- factors based on ALL data will be even larger.

### Fractional atomic coordinates and isotropic or equivalent isotropic displacement parameters (Å^2^)

**Table:**

	*x*	*y*	*z*	*U*_iso_*/*U*_eq_	
Hg1	0.0000	0.19701 (7)	0.2500	0.04474 (19)	
Hg2	0.5000	0.76184 (6)	0.2500	0.04007 (19)	
Cl1	0.13755 (15)	0.0118 (3)	0.36667 (16)	0.0565 (6)	
Cl2	0.35836 (16)	0.9352 (4)	0.22391 (16)	0.0590 (6)	
N1	0.0500 (5)	0.4038 (9)	0.1715 (4)	0.0407 (15)	
N2	0.1527 (6)	0.1618 (12)	0.1736 (6)	0.0497 (17)	
N3	0.4556 (4)	0.5485 (9)	0.1232 (4)	0.0359 (14)	
N4	0.3477 (6)	0.7778 (10)	0.0184 (5)	0.0472 (19)	
C1	0.1163 (5)	0.3496 (11)	0.1495 (5)	0.0362 (16)	
C2	0.1430 (6)	0.4814 (13)	0.1001 (5)	0.0441 (19)	
C3	0.2151 (7)	0.4171 (18)	0.0751 (8)	0.069 (3)	
H3A	0.2217	0.5192	0.0370	0.104*	
H3B	0.1962	0.2928	0.0404	0.104*	
H3C	0.2737	0.3980	0.1318	0.104*	
C4	0.1028 (7)	0.6659 (13)	0.0756 (6)	0.051 (2)	
H4	0.1206	0.7549	0.0432	0.061*	
C5	0.0361 (7)	0.7229 (12)	0.0981 (6)	0.049 (2)	
H5	0.0088	0.8499	0.0813	0.059*	
C6	0.0103 (6)	0.5893 (13)	0.1459 (5)	0.0454 (19)	
H6	−0.0352	0.6267	0.1609	0.055*	
C7	0.3884 (6)	0.5942 (11)	0.0346 (5)	0.0373 (16)	
C8	0.3618 (6)	0.4525 (12)	−0.0402 (5)	0.0403 (17)	
C9	0.2853 (7)	0.5040 (17)	−0.1374 (6)	0.066 (3)	
H9A	0.2783	0.3968	−0.1801	0.100*	
H9B	0.3005	0.6267	−0.1581	0.100*	
H9C	0.2280	0.5215	−0.1364	0.100*	
C10	0.4071 (8)	0.2741 (12)	−0.0193 (6)	0.050 (2)	
H10	0.3904	0.1785	−0.0668	0.060*	
C11	0.4775 (7)	0.2304 (13)	0.0704 (6)	0.049 (2)	
H11	0.5096	0.1087	0.0833	0.058*	
C12	0.4993 (6)	0.3695 (12)	0.1401 (5)	0.0405 (17)	
H12	0.5458	0.3394	0.2012	0.049*	
H2A	0.158 (7)	0.107 (14)	0.224 (4)	0.061*	
H4A	0.301 (5)	0.788 (14)	−0.038 (4)	0.061*	
H2B	0.202 (4)	0.161 (15)	0.168 (7)	0.061*	
H4B	0.348 (7)	0.845 (14)	0.064 (5)	0.061*	

### Atomic displacement parameters (Å^2^)

**Table:**

	*U*^11^	*U*^22^	*U*^33^	*U*^12^	*U*^13^	*U*^23^
Hg1	0.0379 (3)	0.0561 (3)	0.0446 (3)	0.000	0.0238 (2)	0.000
Hg2	0.0326 (3)	0.0476 (3)	0.0330 (3)	0.000	0.0108 (2)	0.000
Cl1	0.0393 (11)	0.0617 (13)	0.0566 (12)	−0.0032 (9)	0.0147 (10)	0.0151 (9)
Cl2	0.0442 (11)	0.0714 (14)	0.0564 (12)	0.0166 (10)	0.0210 (10)	−0.0068 (10)
N1	0.043 (4)	0.041 (4)	0.036 (3)	0.001 (3)	0.018 (3)	0.001 (2)
N2	0.048 (4)	0.051 (4)	0.063 (5)	0.006 (3)	0.037 (4)	0.001 (3)
N3	0.036 (3)	0.036 (3)	0.034 (3)	0.007 (3)	0.016 (3)	0.001 (2)
N4	0.049 (5)	0.039 (4)	0.040 (4)	0.008 (3)	0.012 (3)	0.003 (3)
C1	0.029 (4)	0.046 (4)	0.033 (3)	−0.007 (3)	0.014 (3)	−0.009 (3)
C2	0.045 (5)	0.054 (5)	0.039 (4)	−0.015 (4)	0.025 (4)	−0.009 (3)
C3	0.055 (6)	0.085 (8)	0.081 (7)	−0.007 (5)	0.044 (6)	0.003 (5)
C4	0.065 (6)	0.043 (4)	0.052 (5)	−0.015 (4)	0.034 (4)	−0.003 (4)
C5	0.058 (6)	0.042 (5)	0.042 (4)	0.002 (4)	0.020 (4)	0.002 (3)
C6	0.040 (4)	0.049 (5)	0.039 (4)	0.009 (4)	0.014 (4)	0.001 (3)
C7	0.045 (4)	0.037 (4)	0.030 (3)	−0.006 (3)	0.018 (3)	−0.001 (3)
C8	0.042 (4)	0.049 (4)	0.031 (4)	0.002 (3)	0.019 (3)	0.002 (3)
C9	0.054 (6)	0.087 (7)	0.029 (4)	0.010 (5)	−0.002 (4)	−0.007 (4)
C10	0.066 (6)	0.050 (5)	0.036 (4)	−0.001 (4)	0.027 (4)	−0.009 (3)
C11	0.058 (6)	0.049 (5)	0.045 (5)	0.012 (4)	0.030 (5)	0.004 (3)
C12	0.042 (4)	0.044 (4)	0.032 (3)	0.005 (3)	0.016 (3)	0.004 (3)

### Geometric parameters (Å, º)

**Table:**

Hg1—N1	2.287 (7)	C2—C3	1.497 (14)
Hg1—N1^i^	2.287 (7)	C3—H3A	0.9600
Hg1—Cl1^i^	2.452 (2)	C3—H3B	0.9600
Hg1—Cl1	2.452 (2)	C3—H3C	0.9600
Hg2—N3	2.303 (6)	C4—C5	1.373 (15)
Hg2—N3^ii^	2.303 (6)	C4—H4	0.9300
Hg2—Cl2^ii^	2.446 (2)	C5—C6	1.378 (13)
Hg2—Cl2	2.446 (2)	C5—H5	0.9300
N1—C1	1.356 (11)	C6—H6	0.9300
N1—C6	1.360 (10)	C7—C8	1.425 (10)
N2—C1	1.354 (11)	C8—C10	1.351 (12)
N2—H2A	0.86 (2)	C8—C9	1.498 (11)
N2—H2B	0.87 (2)	C9—H9A	0.9600
N3—C12	1.345 (10)	C9—H9B	0.9600
N3—C7	1.350 (9)	C9—H9C	0.9600
N4—C7	1.352 (10)	C10—C11	1.375 (13)
N4—H4A	0.86 (2)	C10—H10	0.9300
N4—H4B	0.87 (2)	C11—C12	1.365 (12)
C1—C2	1.397 (11)	C11—H11	0.9300
C2—C4	1.355 (13)	C12—H12	0.9300
			
N1—Hg1—N1^i^	106.3 (3)	C2—C3—H3C	109.5
N1—Hg1—Cl1^i^	108.60 (16)	H3A—C3—H3C	109.5
N1^i^—Hg1—Cl1^i^	106.35 (17)	H3B—C3—H3C	109.5
N1—Hg1—Cl1	106.35 (17)	C2—C4—C5	120.7 (8)
N1^i^—Hg1—Cl1	108.60 (16)	C2—C4—H4	119.6
Cl1^i^—Hg1—Cl1	119.88 (11)	C5—C4—H4	119.6
N3—Hg2—N3^ii^	104.2 (3)	C4—C5—C6	119.0 (8)
N3—Hg2—Cl2^ii^	107.38 (18)	C4—C5—H5	120.5
N3^ii^—Hg2—Cl2^ii^	106.20 (16)	C6—C5—H5	120.5
N3—Hg2—Cl2	106.20 (16)	N1—C6—C5	121.3 (9)
N3^ii^—Hg2—Cl2	107.38 (18)	N1—C6—H6	119.4
Cl2^ii^—Hg2—Cl2	123.91 (13)	C5—C6—H6	119.4
C1—N1—C6	119.2 (7)	N3—C7—N4	118.5 (6)
C1—N1—Hg1	123.3 (5)	N3—C7—C8	120.6 (7)
C6—N1—Hg1	117.5 (6)	N4—C7—C8	120.9 (7)
C1—N2—H2A	119 (7)	C10—C8—C7	117.7 (7)
C1—N2—H2B	106 (7)	C10—C8—C9	122.6 (8)
H2A—N2—H2B	116 (10)	C7—C8—C9	119.7 (7)
C12—N3—C7	119.4 (6)	C8—C9—H9A	109.5
C12—N3—Hg2	117.3 (5)	C8—C9—H9B	109.5
C7—N3—Hg2	123.3 (5)	H9A—C9—H9B	109.5
C7—N4—H4A	112 (7)	C8—C9—H9C	109.5
C7—N4—H4B	120 (7)	H9A—C9—H9C	109.5
H4A—N4—H4B	120 (10)	H9B—C9—H9C	109.5
N2—C1—N1	117.8 (7)	C8—C10—C11	121.6 (8)
N2—C1—C2	121.4 (7)	C8—C10—H10	119.2
N1—C1—C2	120.7 (7)	C11—C10—H10	119.2
C4—C2—C1	119.1 (8)	C12—C11—C10	118.6 (8)
C4—C2—C3	121.1 (8)	C12—C11—H11	120.7
C1—C2—C3	119.8 (8)	C10—C11—H11	120.7
C2—C3—H3A	109.5	N3—C12—C11	122.1 (7)
C2—C3—H3B	109.5	N3—C12—H12	118.9
H3A—C3—H3B	109.5	C11—C12—H12	118.9
			
N1^i^—Hg1—N1—C1	152.6 (6)	C1—C2—C4—C5	−0.5 (12)
Cl1^i^—Hg1—N1—C1	−93.3 (5)	C3—C2—C4—C5	179.4 (8)
Cl1—Hg1—N1—C1	37.0 (6)	C2—C4—C5—C6	−0.2 (13)
N1^i^—Hg1—N1—C6	−27.8 (5)	C1—N1—C6—C5	−0.1 (11)
Cl1^i^—Hg1—N1—C6	86.3 (5)	Hg1—N1—C6—C5	−179.8 (6)
Cl1—Hg1—N1—C6	−143.4 (5)	C4—C5—C6—N1	0.5 (13)
N3^ii^—Hg2—N3—C12	−31.0 (5)	C12—N3—C7—N4	−177.6 (8)
Cl2^ii^—Hg2—N3—C12	81.4 (6)	Hg2—N3—C7—N4	2.9 (10)
Cl2—Hg2—N3—C12	−144.2 (6)	C12—N3—C7—C8	1.8 (12)
N3^ii^—Hg2—N3—C7	148.5 (7)	Hg2—N3—C7—C8	−177.7 (6)
Cl2^ii^—Hg2—N3—C7	−99.1 (6)	N3—C7—C8—C10	−1.2 (13)
Cl2—Hg2—N3—C7	35.3 (6)	N4—C7—C8—C10	178.3 (9)
C6—N1—C1—N2	−178.2 (7)	N3—C7—C8—C9	178.3 (8)
Hg1—N1—C1—N2	1.4 (9)	N4—C7—C8—C9	−2.3 (13)
C6—N1—C1—C2	−0.6 (10)	C7—C8—C10—C11	−0.9 (15)
Hg1—N1—C1—C2	179.0 (5)	C9—C8—C10—C11	179.7 (10)
N2—C1—C2—C4	178.4 (8)	C8—C10—C11—C12	2.1 (16)
N1—C1—C2—C4	0.9 (11)	C7—N3—C12—C11	−0.5 (13)
N2—C1—C2—C3	−1.5 (11)	Hg2—N3—C12—C11	179.0 (7)
N1—C1—C2—C3	−179.0 (7)	C10—C11—C12—N3	−1.5 (15)

Symmetry codes: (i) −*x*, *y*, −*z*+1/2; (ii) −*x*+1, *y*, −*z*+1/2.

### Hydrogen-bond geometry (Å, º)

**Table:**

*D*—H···*A*	*D*—H	H···*A*	*D*···*A*	*D*—H···*A*
N2—H2*A*···Cl1	0.86 (2)	2.58 (4)	3.420 (9)	164 (10)
N2—H2*B*···Cl2^iii^	0.87 (2)	2.72 (7)	3.420 (8)	139 (8)
N4—H4*A*···Cl1^iv^	0.86 (2)	2.70 (7)	3.409 (8)	140 (9)
N4—H4*B*···Cl2	0.87 (2)	2.59 (4)	3.424 (9)	162 (9)

Symmetry codes: (iii) *x*, *y*−1, *z*; (iv) *x*, −*y*+1, *z*−1/2.
